# The NLRP3 Inflammasome and Its Role in T1DM

**DOI:** 10.3389/fimmu.2020.01595

**Published:** 2020-08-27

**Authors:** Xiaoxiao Sun, Haipeng Pang, Jiaqi Li, Shuoming Luo, Gan Huang, Xia Li, Zhiguo Xie, Zhiguang Zhou

**Affiliations:** ^1^Department of Metabolism and Endocrinology, The Second Xiangya Hospital, Central South University, Changsha, China; ^2^Key Laboratory of Diabetes Immunology (Central South University), Ministry of Education, National Clinical Research Center for Metabolic Diseases, Changsha, China

**Keywords:** T1DM, NLRP3 inflammasome, IL-1β, innate immunity, autoimmune disorder

## Abstract

The NLRP3 (nucleotide-binding and oligomerization domain-like receptor family pyrin domain-containing 3) inflammasome is a protein complex expressed in cells. It detects danger signals and induces the production of active caspase-1 and the maturation and release of IL (interleukin)-33, IL-18, IL-1β and other cytokines. T1DM (type 1 diabetes mellitus) is defined as a chronic autoimmune disorder characterized by the autoreactive T cell-mediated elimination of insulin-positive pancreatic beta-cells. Although the exact underlying mechanisms are obscure, researchers have proposed that both environmental and genetic factors are involved in the pathogenesis of T1DM. Furthermore, immune responses, including innate and adaptive immunity, play an important role in this process. Recently, the NLRP3 inflammasome, a critical component of innate immunity, was reported to be associated with T1DM. Here, we review the assembly and function of the NLRP3 inflammasome. In addition, the activation and regulatory mechanisms that enhance or attenuate NLRP3 inflammasome activation are discussed. Finally, we focus on the relationship between the NLRP3 inflammasome and T1DM, as well as its potential value for clinical use.

## Introduction

The inflammatory response is a common mechanism of many diseases. However, the clinical manifestations caused by the combination of a certain microenvironment and a variety of stimuli from common or specific pathways are different. Currently, many chronic diseases, particularly diabetes, are serious threats to human health. Many researchers have recognized that inflammatory immune factors induce many chronic diseases. Innate immune cells induce a series of inflammatory responses by detecting various PAMPs (pathogen-associated molecular patterns) or DAMPs (damage-associated molecular patterns) through innate sensors (1). With a relative molecular mass of approximately 700,000 Da, the NLRP3 inflammasome is a polyprotein complex that plays a critical role in the course of inflammatory responses (2). The NLRP3 inflammasome is comprised of NLRP3, ASC (apoptosis-associated speck-like protein containing a caspase recruitment domain), and procaspase-1 (3, 4). It is the most well-studied inflammasome and functions as a site for the activation of caspase-1 (3, 5). Based on emerging evidence, activated caspase-1 causes the maturation of IL-1 (6, 7). Because the NLRP3 inflammasome may trigger the release of IL-1β after stimulation with various danger signals, it represents a potentially effective target to regulate the onset and development of various autoimmune diseases, such as T1DM.

T1DM is defined as an organ-specific autoimmune disorder characterized by the autoreactive T cell-mediated elimination of insulin-producing pancreatic beta-cells (8). Although the exact underlying mechanisms are still unknown, a combination of environmental and genetic elements are involved in the pathophysiological process of T1DM (9–11). Both innate immunity and adaptive immunity are involved in the progression of T1DM (12–14). Innate immunity, which serves as the first line of defense against an exogenous attack by bacteria, viruses, and fungi, is a relatively conserved immune response compared with adaptive immunity (15, 16). Previous studies have confirmed that the innate immune system exerts its effect *via* highly conserved PRRs (pattern-recognition receptors) to initiate innate inflammatory responses to both exogenous and endogenous trigger factors and further activate adaptive immunity (16–18). Upon the recognition of DAMPs and PAMPs, which are associated with cellular stress and microbial pathogens, PRRs promote the secretion of proinflammatory cytokines by inducing either non-transcriptional or transcriptional innate immune responses (19, 20). NLRP3 is a PRR, and the NLRP3 inflammasome is a component of the innate immune system that plays a key role in the inflammatory response. In this review, we discuss the components and functions of the NLRP3 inflammasome and the activation mechanisms and regulatory mechanisms that potentiate or limit NLRP3 inflammasome activation. In addition, we describe the function of the NLRP3 inflammasome in T1DM to provide a potential treatment target for the prevention and improvement of this disorder.

## Components and Function of the NLRP3 Inflammasome

**FIGURE 1:**
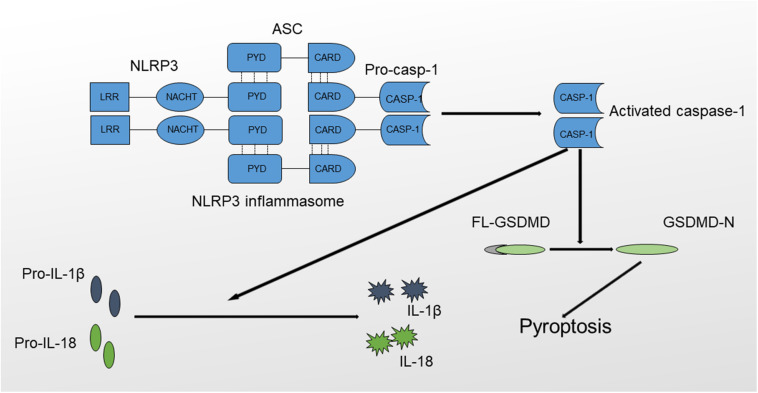
Structure and function of the NLRP3 inflammasome. The NLRP3 inflammasome comprises NLRP3, ASC and procaspase-1. The formation of the NLRP3 inflammasome results in the activation of caspase-1 through the self-cleavage of procaspase-1. Activated caspase-1 causes the maturation of IL-1β and IL-18 and triggers inflammatory cell death mediated by GSDMD, also termed pyroptosis.

The NLRP3 inflammasome is a protein complex that includes procaspase-1, ASC and NLRP3 (21). NLRP3 is a member of the NLR (Nod like-receptor) protein family, which is widely expressed in macrophages, monocytes, and dendritic cells and has the function of recognizing pathogens. NLRP3 has a characteristic NLR protein family LRR (leucine-rich repeat) domain at the C-terminus (22). The middle region of NLRP3 is called the NBD (nucleotide-binding domain), also known as NOD or NACHT. The NBD belongs to the NTPase superfamily and hydrolyzes ATP into GTP. The N-terminus contains a PYD (pyrin domain), which is also called the CARD (caspase recruitment domain) or BIR (baculovirus IAP repeat) domain; this domain participates in multiple inflammatory responses by binding molecules with the same domain. For example, ASC is bound *via* the PYD-PYD interaction. ASC is the adapter protein of the NLRP3 inflammasome. The N-terminus of ASC contains a PYD domain that is the same as the PYD domain in NLRP3, whereas the C-terminus contains a CARD recruitment domain that is the same as the CARD domain in procaspase-1. Therefore, ACS functions as a dual adapter protein molecule that binds to both NLRP3 and procaspase-1 through PYD-PYD and CARD-CARD domain interactions. Caspase-1, also called IL-1β-converting enzyme, is an effector protein of the inflammasome that cleaves inactive proinflammatory cytokines, including pro-IL-1β and pro-IL-18, to produce activated IL-1β and IL-18 (Figure 1) (23, 24).

NLRP3 activation results in the oligomerization and recruitment of ASC and procaspase-1, which increase the autocleavage and maturation of procaspase-1. Active caspase-1 cleaves pro-IL-1β to produce mature IL-1β, which, when released, recruits other inflammatory cells and exerts direct cytotoxic effects. In addition, the NLRP3 inflammasome mediates a special type of programmed cell death named pyroptosis, which is inherently inflammatory and is triggered by pathological stimuli through the activation of caspase-1 (25, 26). The process of pyroptosis is mediated by Gasdermin (GSDMD), consisting of an amino-terminal cell death region, a carboxy-terminal autoinhibitory region, and a central linker domain (27). GSDMD is activated by caspase-1 through the removal of its carboxyl inhibitory terminus, and activated GSDMD induces cell death characterized by plasma membrane rupture, DNA cleavage and cell lysis by binding to the inner leaflet of the cell membrane, oligomerizing and forming a pore containing 16 symmetrical promoters (28). Based on the results of *in vitro* studies, activated GSDMD possesses a bactericidal property, but the exact mechanisms remain obscure (29). In addition, GSDMD-dependent pyroptosis promotes IL-1β and IL-18 release *via* a non-conventional pathway (30, 31). In conclusion, caspase-1 activation will result in the production of activated proinflammatory cytokines and lead to rapid cell death (32, 33).

NLRP3 is activated by a number of pathogens, as well as many PAMPs and DAMPs, which are structurally diverse, and environmental irritants. NLRP3 oligomerizes *via* homotypic interactions between NACHT domains to form a high-molecular-weight complex that triggers procaspase-1 activation when it is stimulated (22). The pathogenic agents that activate the NLRP3 inflammasome include (1) the fungi *Saccharomyces cerevisiae* and *Candida albicans* that function *via* the Syk signaling pathway (34); (2) a pore-forming toxin-producing bacteria (35); and (3) viruses, including the influenza virus, adenovirus, and the Sendai virus (36, 37).

## Mechanisms Underlying the Activation and Regulation of the NLRP3 Inflammasome

### Mechanisms Underlying the Activation of the NLRP3 Inflammasome

The NLRP3 inflammasome is activated by a wide range of stimuli. For example, the NLRP3 inflammasome detects signals produced by metabolism, such as increased extracellular glucose levels, which is an essential manifestation of diabetes (38). However, given their structural and chemical dissimilarity, NLRP3 is not likely to be activated through a direct interaction with its stimuli (39). Researchers have speculated that different agonists will lead to a common cellular event that ultimately activates the NLRP3 inflammasome.

When NLRP3 is not activated, the LRR domain interacts with HSP90 (heat-shock protein 90) and the ubiquitin ligase-associated protein SGT1HSP90, which are likely to maintain NLRP3 in an inactive but signaling-competent state (40). Two types of signals are needed to activate the NLRP3 inflammasome (41). First, a ligand binds to TLR4 on the membrane to provide the first signal that induces the expression of NLRP3, IL-1β, and IL-18 by triggering the NF-kB signaling pathway (42). In addition, TLR4 may provide the first signal *via* an unknown mechanism through the proteins myD88 and IRAK1. A low level of TLR4 stimulation is sufficient for the ATP activation pathway, and this pathway does not require the synthesis of new proteins (43–45). Because the expression of endogenous NLRP3 in immune cells is not sufficient to activate the NLRP3 inflammasome, the activation of NF-kB is necessary for the sufficient production of NLPR3. The second signal is the appearance of the activator of the NLRP3 inflammasome. The NLRP3 inflammasome begins to assemble when it is stimulated (46).

The mechanisms underlying the activation of the NLRP3 inflammasome are still not completely understood and may be associated with ROS (reactive oxygen species) production, lysosomal damage, P2X7R (purinergic ligand-gated ion channel 7 receptor) activation, and K^+^ efflux. To date, three models explaining the activation of the NLRP3 inflammasome have been acknowledged by most researchers.

#### K^+^ Efflux

The first model concerns the efflux of K^+^, which is the most common mechanism of NLRP3 inflammasome activation. A decreasing cytosolic level of K^+^ induced by NLRP3 stimuli, ATP, or nigericin mediates IL-1β activation and release in mouse macrophages and human monocytes (47, 48). Moreover, the efflux of K^+^ alone results in the activation of NLRP3, and a high extracellular K^+^ concentration inhibits NLRP3 activation (49). Therefore, the intracellular hypokalemia that induces mitochondrial damage and the subsequent release of ROS and mtDNA (mitochondrial DNA) is sufficient to activate the NLRP3 inflammasome (49, 50). In addition, K^+^ efflux is necessary to activate NLRP3 in caspase-11-mediated non-canonical inflammasome signaling (6, 51).

Three explanations for K^+^ efflux have been proposed. First, bacterial toxins destroy the integrity of the cell membrane, and thus K^+^ flows out along the ion concentration gradient (52). Second, the combination of extracellular ATP and the pyrogenic P2X7 ATP-gated ion channel (53) triggers K^+^ efflux. Another type of channel, pannexin-1, also participates in the activation of NLRP3 *via* other ATP-dependent pathways (54–56). Third, microbial molecules are delivered to the cytosol in a pannexin 1-independent manner (54). The generation of pores disrupts the intracellular K^+^ concentration gradient and transports bacterial molecules to the cytosol, which may help clarify how bacteria that do not exist in the cytosol activate cytosolic sensors.

Although K^+^ efflux has been considered the most common mechanism to activate the NLRP3 inflammasome, recent reports have indicated that some small molecules, including CL097 and GB111-NH_2_, activate NLRP3 independently of K^+^ efflux (57). Moreover, an *NLRP3* mutant leads to inflammasome activation induced by lipopolysaccharides in the absence of K^+^ efflux in mouse macrophages (58). In conclusion, K^+^ efflux is sufficient, but not unique, in activating this inflammasome. Further investigations are needed to elucidate the underlying mechanisms by which NLRP3 senses alterations in the intracellular K^+^ concentration.

#### Lysosomal Damage

The second model concerns lysosomal damage. Particulate matter, such as MSU, and adjuvants including alum (59, 60) activate the NLRP3 inflammasome in macrophages. The phagocytosis of specific particulate structures and crystalline structures results in lysosomal membrane disintegration and damage and the cytosolic release of lysosomal contents, which are sensed by the NLRP3 inflammasome to some extent.

Lysosomal disruption triggered by Leu-Leu-OMe activates the NLRP3 inflammasome (61). However, the exact mechanisms by which lysosomal damage contributes to the activation of NLRP3 remain obscure. Currently, two factors, lysosomal acidification and cathepsins, have been identified to be associated with the activation mechanisms. An H^+^ ATPase inhibitor blocks NLRP3 inflammasome activation induced by particulate matter in macrophages (61). Additionally, both *in vitro* and *in vivo* experiments suggest that inhibitors of lysosomal acidification suppress IL-1β production (62). In fact, the acidic conditions tend to cause Na^+^ release and increase cellular osmolarity and subsequent water influx, resulting in intracellular hypokalemia (62). Moreover, lysosomal rupture leads to enzyme release and the activation of the NLRP3 inflammasome. These proteases suppress the activation of negative regulators and increase the activation of NLRP3 through proteolytic reactions, which lead to inflammasome assembly (63). Lysosomal protease CTSB (cathepsin B) plays an important role in the model. CTSB inhibitors attenuate NLRP3 activation in macrophages treated with particulate matter (6). Furthermore, lysosomal CTSB release is required for IL-1β secretion, indicating the participation of CTSH in NLRP3 activation (64). Therefore, the cytosolic release of lysosomal contents is another mechanism of NLRP3 inflammasome activation. However, CTSB-deficient mouse macrophages show normal caspase-1 activation and IL-1β maturation induced by particulate NLRP3 agonists, suggesting that some undefined mechanisms exist (65). Further research is required to solve the existing conflicts and clarify the actual role of lysosomal damage in NLRP3 inflammasome activation.

#### Reactive Oxygen Species and Mitochondrial Dysfunction

The third model concerns the generation of ROS. In this model, all agonists of NLRP3 induce ROS production, and this collective pathway involves the NLRP3 inflammasome (66–68). All NLRP3 agonists that have been confirmed, including particulate activators and ATP, induce ROS production, and chemical scavengers that block ROS generation inhibit inflammasome activation (34, 66–69). Consistent with the role of ROS production, the activation of caspase-1 by asbestos is suppressed in NAC (*N*-acetyl cysteine)-treated cells, in which NAC inhibits ROS generation (67). The source of ROS is currently unknown, but NADPH oxidases may be associated with their production, as *in vitro* studies indicate that inhibition of the common p22 subunit, which plays a critical role in ROS formation, suppresses inflammasome activation (67). However, the genetic or pharmacological blockade of NADPH oxidase does not affect NLRP3 activation in both mouse and human cells. The tissue-specific role of ROS may explain the differences in the activation of NLRP3 inflammasome. NOX2 (NADPH oxidase 2) knockout mice were recently shown to display decreased production, assembly and activation of the NLRP3 inflammasome in the injured cerebral cortex, but not in the umbilical vein endothelium.

The mechanisms underlying ROS-dependent NLRP3 inflammasome activation remain to be revealed in more detail. Recently, a ROS-sensitive NLRP3 ligand, TXNIP/VDUP1 (thioredoxin-interacting protein), was shown to be involved in NLRP3 activation (38, 70). When cellular phagocytosis is dysfunctional, activators such as uric acid crystals increase ROS production and simultaneously trigger the dissociation of TXNIP from TRX (thioredoxin). TXNIP has been identified as a common binding partner of TRX (71). TXNIP decreases the reductase activity of TRX by directly interacting with the redox-active part of TRX. A yeast two-hybrid screen using the LRRs of NLRP3 as bait revealed that TXNIP is also a potential binding partner of NLRP3 (72, 73). Overexpressed TXNIP and endogenous TXNIP interact with the LRR region of NLRP3, and the nucleotide-binding NACHT domain of NLRP3 also interacts with TXNIP. NLRP3 detects the presence of ROS, the production of which in cells is directly or indirectly induced by activators of the NLRP3 inflammasome. The complex formed by TXNIP and TRX senses increasing amounts of ROS and causes the dissociation of the complex. Subsequently, the interaction of TXNIP and NLRP3 activates NLRP3, recruits ASC and procaspase-1, and leads to the assembly of the active NLRP3 inflammasome. Intriguingly, accumulating evidence indicates that TXNIP is associated with glucose metabolism and diabetes (74). In pancreatic beta-cells, the expression of TXNIP is downregulated by insulin and is consistently increased in patients diagnosed with T2DM (type 2 diabetes mellitus) (74). Additionally, mutations in TXNIP are associated with reduced plasma glucose levels and hypertriglyceridemia (75). Published data that have been confirmed suggest that the expression of TXNIP is substantially upregulated by exposure to high glucose concentrations in pancreatic islet cells (76, 77). Although the ROS model is supported by many studies, many questions still remain and need to be resolved. For example, researchers have not clarified whether the mechanism by which superoxide directly inhibits caspase-1 activity by regulating redox-sensitive cysteines (78) provides dose- or temporal-dependent negative feedback to limit the function of caspase-1 triggered by a ROS-dependent NLRP3 pathway.

In recent years, mitochondria have been shown to play an essential role in the activation of the NLRP3 inflammasome (79, 80). Mitochondria are an ideal platform to assemble the NLRP3 inflammasome. On the other hand, NLRP3 is directly affected by molecules from mitochondria, such as mitochondrial ROS (mtROS), mtDNA, and cardiolipin.

### Negative Regulatory Mechanisms of the NLRP3 Inflammasome

NLRP3 promotes the secretion of IL-33, IL-18, and IL-1β, which are very important molecules that control pathological infections. However, the excessive production of cytokines exerts a deleterious effect on the body. For instance, the excessive activation of proinflammatory cytokines, including TNF-α, IL-1β, and IFNs, is associated with autoimmune diseases, such as T1DM. Therefore, the activation of the NLRP3 inflammasome must be strictly regulated to maintain the balance of the internal environment and homeostasis. Four negative regulatory mechanisms of the NLRP3 inflammasome have been identified.

#### Negative Regulatory Molecules

The first mechanism is associated with negative regulatory molecules. A group of small proteins that consist of either a PYD or a CARD domain have emerged as key regulators of the inflammasome. As two types of endogenous dominant-negative proteins, both COPs (CARD-only proteins) and POPs (PYD-only proteins) decrease the activity of the NLRP3 inflammasome in response to tissue injury and pathogen infection (81).

POPs, such as POP1 and POP2, which display 64 and 37% homology with the PYD subunit of ASC, respectively, prevent ASC recruitment to NLRP3 by interacting with ASC in a PYD-dependent manner and replacing other proteins that interact with ASC (82). *In vitro* overexpression models confirm that POP1 and POP2 bind to ASC and block the interaction between NLRP3 and ASC (83). Moreover, *in vivo* studies using transgenic mice expressing POP2 have revealed decreased inflammatory cytokine levels in response to LPS, and the animals tend to resist bacterial infections compared with wild-type mice (84). To date, five proteins belonging to the COP family have been identified, including Iceberg, Nod2-S, caspase-12s, COP1/pseudo-ICE and INCA (27). COPs, which are extremely similar to the CARD subunit of procaspase-1, function as decoy proteins by isolating caspase-1 through CARD domain interactions and preventing its binding to activating adaptors (83). Because the expression of Iceberg is increased in the inflammatory environment, this protein appears to function as a negative feedback regulator that inhibits systemic inflammation. Notably, our understanding of the regulatory effects of POPs and COPs on NLRP3 activation is limited because these molecules are not expressed in mice. However, the development of transgenic mice provides a great opportunity to further analyze these proteins.

#### Cells and Cytokines

The second mechanism involves certain cells and cytokines. Various immunocytes and proinflammatory cytokines participate in the downregulation of inflammasome activation. For example, human-derived activated memory T cells negatively regulate the P2X7R signaling pathway, leading to the inhibition of the NLRP3 inflammasome (85). C5aR2 (C5a receptor 2), which is expressed on the T cell surface, inhibit NLRP3 inflammasome assembly by inversely modulating C5 activation and stimulating C5aR1 (C5a receptor 1) (85, 86). IL-1β signaling promotes the recruitment of neutrophils; in turn, increased neutrophil apoptosis results in the resolution of the inflammatory response (87). The type I interferon signaling pathway represses the activity of the NLRP3 inflammasome and inhibits the maturation of IL-1β through the STAT1 transcription factor (88).

#### Autophagy

The third mechanism is associated with autophagy. Autophagy, also referred to as macroautophagy, is a conserved process involving the transport of the cytoplasmic content to lysosomes *via* autophagosomes, and the substrates degraded by this process mainly include long-lived proteins, intracellular pathogens and organelles. Autophagy is involved in the pathogenesis of many inflammatory disorders and modulates many aspects of the immune response, including inflammation (89, 90).

Autophagy inhibits the formation of the NLRP3 inflammasome by degrading impaired mitochondria, decreasing mtROS production and disaggregating the ASC complex (91). *In vitro* experiments using macrophages from mouse models indicate that the depletion of beclin 1 and LC3B, proteins that are associated with autophagy, increases the activation of caspase-1 and release of IL-18 and IL-1β by impairing mitochondrial homeostasis (50, 92, 93). Additionally, autophagy dysfunction, whether it is caused by autophagy deficiency or mitochondrial inhibitors, increases the activation of the NLRP3 inflammasome via the production of mtROS (92). Consistent with the result obtained from cell-based experiments, *in vivo* studies suggest that LC3B-dificient mice produce caspase-1-depedent cytokines at higher levels and exhibit a greater susceptibility to LPS (92). Therefore, autophagy is closely associated with the well-being of mitochondria, and autophagy and the mitochondria modulate the NLRP3-dependent inflammatory response together.

#### NO and CO

Finally, the other mechanisms, including the production of NO (nitric oxide) and CO (carbon monoxide), negatively regulate the NLRP3 inflammasome. NO regulates multiple physiological responses and defends against pathogens. Endogenous NO downregulates NLRP3 inflammasome activation and stabilizes mitochondria. According to the results of *in vitro* experiments, NO blocks NLRP3-mediated caspase-1 and IL-1β activation in mice and human myeloid cells (94). Additionally, decreased production of NO caused by iNOS (inducible NO synthase) elimination or pharmacological blockade leads to increased cytokine production induced by the activated NLRP3 inflammasome and the accumulation of dysfunctional mitochondria *in vivo* (94). CO is toxic and damages the respiratory system. However, endogenous CO possesses anti-inflammatory properties. CO inhibits the production of IL-1β induced by inflammasomes and suppresses the activation of the NLRP3 inflammasome in bone marrow-derived macrophages; furthermore, CO inhibits mtROS generation, preserves the integrity of mitochondrial membrane, and prevents mtDNA translocation into cytosol (95, 96). Therefore, we conclude that NO and CO negatively modulate the activation of the NLRP3 inflammasome mainly by stabilizing mitochondria.

## The NLPR3 Inflammasome and T1DM

**FIGURE 2:**
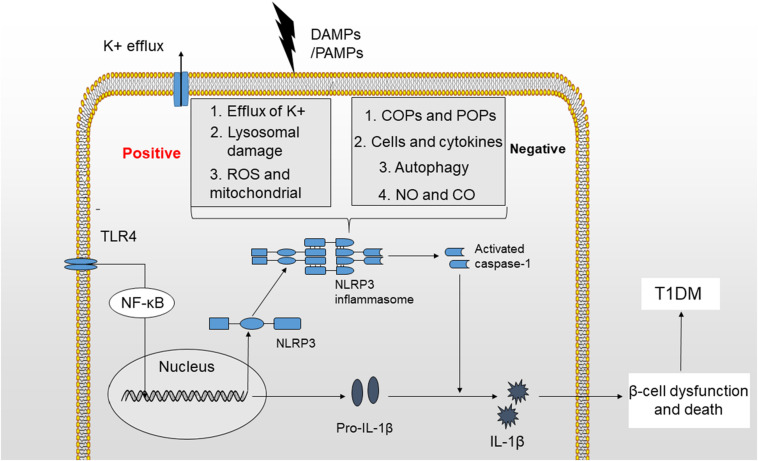
Mechanisms of NLRP3 inflammasome activation. The activation of the NLRP3 inflammasome requires two signals. (1) TLR4 stimulation increases the production of NLRP3 and pro-IL-1β by activating the NF-κB signaling pathway. (2) K^+^ efflux, cathepsin release by ruptured lysosomes and ROS generation provide a second signal that may activate the NLRP3 inflammasome and produce activated caspase-1, leading to the maturation of IL-1β. The activation of NLRP3 inflammasome is potentially negatively regulated by small molecules, such as COPs and POPs, cells and cytokines, autophagy, NO, and CO. Finally, IL-1β induces beta-cell dysfunction and death, which will ultimately lead to the development of T1DM.

The NLRP3 inflammasome has consistently been shown to participate in the pathogenesis of many autoimmune disorders, including MS (multiple sclerosis), EAE (experimental autoimmune encephalomyelitis), IBD (inflammatory bowel disease) and T1DM (97–100). T1DM is a metabolic disease characterized by an absolute deficiency in insulin and subsequent hyperglycemia resulting from an autoimmune assault. Autoreactive T cells infiltrate pancreatic islets and induce insulitis, causing beta-cell death (101). In addition to adaptive immunity, innate immunity plays an important role in the pathogenesis of T1DM. Based on accumulating evidence, among all components of the innate immune system, the NLRP3 inflammasome and its downstream cytokines, particularly IL-1β, are involved in the development of T1DM (Figure 2) (102, 103).

IL-1β induces the migration of proinflammatory cells into pancreatic islets, mediates cytokine-induced beta-cell apoptosis, exerts direct cytotoxic effects on beta-cells, and may function as inflammatory signal in the early stage of T1DM (102, 104, 105). IL-1β levels are increased in both patients with a new T1DM diagnosis and patients with chronic T1DM compared with healthy controls, and after treatment, IL-1β levels are decreased in patients who have been newly diagnosed with T1DM (106–108). Furthermore, the levels of IL-1 receptor antagonist (IL-1RA), which inhibits the interaction between IL-1β and its receptor and blocks downstream signaling, are decreased in islets from non-diabetic donors exposed to sera from patients with T1DM, and decreased expression of IL-1RA not only results in insulin-producing beta-cell dysfunction and death but also IL-1β production, thus further affecting beta-cells (109). Additionally, NOD mice pretreated with IL-1RA exhibit reduced chemical-induced hyperglycemia, but not islet inflammation (110). Based on these findings, some new treatment strategies aiming to suppress IL-1β activity, such as synthetic IL-1RA and IL-1β traps, have been developed to reverse or ameliorate autoimmune diseases. Indeed, patients with T1DM have decreased requirements for insulin and similar HbA1c (hemoglobin A1c) levels after IL-1RA treatment (102, 111). However, some discrepancies remain to be solved. Animal experiments with NOD caspase-1^(–/–)^ mice revealed reduced IL-1 levels, but an unchanged incidence of diabetes and sensitivity to streptozotocin compared with wild type NOD mouse models (112). At a minimum, caspase-1-mediated IL-1β production is not indispensable for diabetes development in NOD mice. Another *in vivo* experiment also indicates that CD4 + T cell-induced beta-cell death and diabetes is independent of IL-1 and IL-18 in NOD mice (113). Moreover, the larger, multicenter preclinical studies did not observe synergistic effects of IL-1 blockade and anti-CD3 therapy on new-onset autoimmune diabetes in NOD mouse models (114). In conclusion, the role of IL-1β in T1DM pathogenesis is not completely understood and further research is required before its clinical application.

Because IL-1β exerts a systemic effect on immunological intolerance and plays a potential role in T1DM development, its upstream activator, the NLRP3 inflammasome, is a functionally plausible complex contributing to the pathogenesis of T1DM. that the expression of the NLRP3 inflammasome in human pancreatic islets is regulated by LPS (115). Interestingly, recent genetic association studies have indicated a potential association between polymorphisms in inflammasome genes and an increased risk of developing T1DM. As shown in our previous study, SNPs (single-nucleotide polymorphisms) in the NLRP1 gene are associated with T1DM, as well as the age of onset in Chinese Han patients with T1DM (100). More importantly, two SNPs within NLRP3 were found to be associated with an increased risk of T1DM and celiac disease in a separate study. An association between T1DM and a risk SNP (rs10754558) within NLRP3 in the northeastern Brazilian population was identified in a human study (116). However, the contribution of this mutation to the genetic predisposition should be further confirmed in other populations and its resulting function requires further study. In addition, the diabetogenic role of NLRP3 has been observed in animal experiments. NLRP3 was recently shown to play an important role in the immune development of T1DM in NOD mice (103). NLRP3 deficiency affects the activation and maturation of T cells, and more importantly, the elimination of NLRP3 alters the migration of T cells to pancreatic islets, which is a critical pathogenic process that causes beta-cell damage. Furthermore, NLRP3 knockout downregulates the expression of the chemokines CCL5 (C-C motif ligand 5) and CXCL10 (C-X-C motif ligand 10) in pancreatic islet cells in an IRF-1-dependent manner, suggesting that it regulates chemotaxis (103). Moreover, mtDNA-mediated NLRP3 activation induces caspase-1-dependent IL-1β production and contributes to STZ (streptozotocin)-induced T1DM in a murine model, directly indicating a diabetogenic effect of NLRP3-caspase-1-IL-1β signaling (117). Recently, mtDNA was also shown to be involved in the vascular dysfunction in individuals with T1DM, highlighting the association of the NLRP3 inflammasome with diabetic complications (118). Other studies have confirmed that glibenclamide and metformin, both of which are typical treatments for diabetes, have the potential to inhibit the activation of the NLRP3 inflammasome, indirectly indicating that the NLRP3 inflammasome is associated with T1DM. In summary, all of the evidence mentioned above indicates a close relationship between T1DM and the NLRP3 inflammasome.

However, some questions regarding the NLRP3 inflammasome remain to be explored and resolved. The inflammasome exerts a protective effect on maintaining immune homeostasis (119–121). More importantly, the expression of the NLRP3 inflammasome is downregulated in patients with SLE compared with healthy controls and is negatively correlated with disease activity, indicating a protective effect of the inflammasome on SLE (122). Consistent with these findings, another study examining T1DM also indicated that downregulated NLRP3 inflammasome signaling participates in the early stage of autoimmune diabetes (123). Further studies are required to investigate whether the downregulation of the NLRP3 inflammasome is an outcome or cause of the progression of autoimmune disorders. Moreover, some inflammasome-independent pathways activate IL-1β and are potentially involved in development of T1DM (124, 125), indicating that the onset of T1DM is caused by complex networks rather than a single pathway. In summary, a better understanding of the NLRP3 inflammasome is still needed to completely ascertain its effect on the pathogenesis of T1DM and develop new treatment strategies.

## Conclusion and Perspectives

Researchers have improved their knowledge of the NLRP3 inflammasome. We have identified many different activators and a close relationship with inflammatory diseases, including T1DM. However, the regulatory mechanisms and their function in the development of the disease must be further clarified. The discovery of the NLRP3 inflammasome has provided a new opportunity to explore the pathogenesis of inflammation-related diseases. In-depth research into the NLRP3 inflammasome, which is a regulator of IL-18 and IL-1β, will provide a new strategy for the treatment and prevention of inflammatory diseases.

Currently, the pathogenesis of T1DM is not completely understood. However, both environmental and genetic factors are involved in the onset and development of T1DM. The overactivation of the immune system, including innate immune responses, resulting from predisposing genetic mutations is the main cause of the loss and dysfunction of beta-cells. The NLRP3 inflammasome is much more likely to predispose an individual to the onset of T1DM. However, the mechanisms of NLRP3 inflammasome activation and regulation remain obscure, and the exact role of this inflammasome in the pathogenesis of T1DM requires further investigation. We propose that immunotherapy targeting the NLRP3 inflammasome is a promising approach to fight T1DM.

## Author Contributions

XS performed the literature search and wrote the first draft of the manuscript. HP generated the figures and revised the text. JL, SL, GH, and XL critically revised the text and provided substantial scientific contributions. ZZ and ZX proposed the project and revised the manuscript. All authors approved the final version of the manuscript.

## Conflict of Interest

The authors declare that the research was conducted in the absence of any commercial or financial relationships that could be construed as a potential conflict of interest.
